# Examining strategies to facilitate vitamin B_1_ biofortification of plants by genetic engineering

**DOI:** 10.3389/fpls.2013.00160

**Published:** 2013-05-29

**Authors:** Lucille Pourcel, Michael Moulin, Teresa B. Fitzpatrick

**Affiliations:** Department of Botany and Plant Biology, University of GenevaGeneva, Switzerland

**Keywords:** thiamin, riboswitch, HMP, HET, biofortification, genetic engineering

## Abstract

Thiamin (vitamin B_1_) is made by plants and microorganisms but is an essential micronutrient in the human diet. All organisms require it as a cofactor in its form as thiamin pyrophosphate (TPP) for the activity of key enzymes of central metabolism. In humans, deficiency is widespread particularly in populations where polished rice is a major component of the diet. Considerable progress has been made on the elucidation of the biosynthesis pathway within the last few years enabling concrete strategies for biofortification purposes to be devised, with a particular focus here on genetic engineering. Furthermore, the vitamin has been shown to play a role in both abiotic and biotic stress responses. The precursors for *de novo* biosynthesis of thiamin differ between microorganisms and plants. Bacteria use intermediates derived from purine and isoprenoid biosynthesis, whereas the pathway in yeast involves the use of compounds from the vitamin B_3_ and B_6_ groups. Plants on the other hand use a combination of the bacterial and yeast pathways and there is subcellular partitioning of the biosynthesis steps. Specifically, thiamin biosynthesis occurs in the chloroplast of plants through the separate formation of the pyrimidine and thiazole moieties, which are then coupled to form thiamin monophosphate (TMP). Phosphorylation of thiamin to form TPP occurs in the cytosol. Therefore, thiamin (or TMP) must be exported from the chloroplast to the cytosol for the latter step to be executed. The regulation of biosynthesis is mediated through riboswitches, where binding of the product TPP to the pre-mRNA of a biosynthetic gene modulates expression. Here we examine and hypothesize on genetic engineering approaches attempting to increase the thiamin content employing knowledge gained with the model plant *Arabidopsis thaliana*. We will discuss the regulatory steps that need to be taken into consideration and can be used a prerequisite for devising such strategies in crop plants.

## INTRODUCTION

Vitamin B_1_, also known as thiamin, was the first B vitamin to be identified (Funk, 1912). In cells, it exists as three predominant vitamers, i.e., free thiamin, thiamin monophosphate (TMP), and thiamin pyrophosphate (TPP), although adenylated and triphosphorylated forms also exist (Bettendorff et al., 2007; Gangolf et al., 2010). The cofactor form of the vitamin is TPP, which is essential for the activity of enzymes involved in acetyl-CoA and amino acid biosynthesis, as well as in the Krebs and Calvin cycles. Acute deficiency in vitamin B_1_ in humans leads to a disease called beriberi, which can result in fatal neurological and cardiovascular disorders. Therefore, vitamin B_1_ as an essential micronutrient for humans must be taken in the diet. Free thiamin is the predominant B_1_ vitamer used and can be taken up *via* specific carriers in epithelial cells (Komai et al., 1974; Said et al., 1999). Paradoxically, although the main source of vitamin B_1_ in the diet is plants, the edible portions of some of the most abundant crops used in human nutrition have a natural thiamin content below the recommended dietary allowance (RDA, 1.3 mg/day), e.g., rice (*Oryza sativa*, 18% RDA), wheat (*Triticum aestivum*, 25% RDA), and maize (*Zea mays*, 33% RDA; Fitzpatrick et al., 2012). In addition to a low content in thiamin, crop postharvest processing can aggravate this shortfall. Moreover, thiamin is unevenly distributed in cereal grains. The aleurone layer and germ, which are removed during the refining process, are much richer than the endosperm. Therefore, most of the thiamin is lost in the production of white flour and polished rice. To overcome this problem, wheat flour and rice are often supplemented with chemically synthesized thiamin in developed countries. However, although being common and/or compulsory (in the case of enriched wheat flour in certain developed countries; Harper et al., 1998), such postharvest supplementation remains expensive and inaccessible to people in developing countries, where crops represent the most important dietary source of vitamin B_1_. An alternative approach to tackle the problem of malnutrition is to improve crop nutritional qualities through biofortification using genetic engineering or conventional breeding.

**FIGURE 1:**
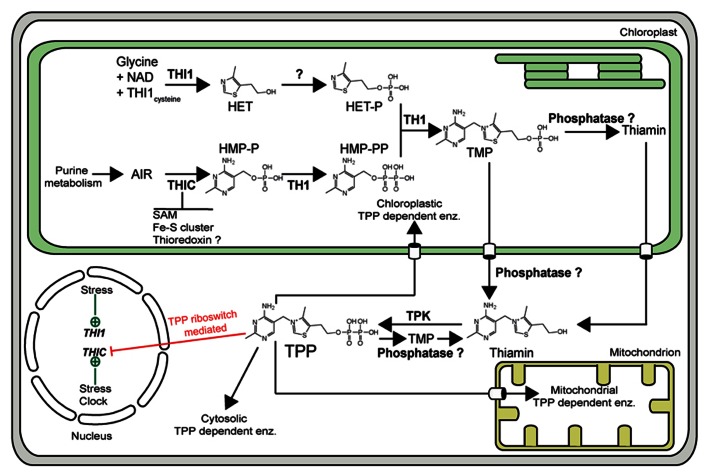
**The thiamin biosynthesis pathway of *Arabidopsis thaliana***. As in all organisms, TMP is generated from the condensation of HET-P and HMP-PP by the action of TMP synthase (TH1). THI1 catalyzes the formation of the thiazole moiety from NAD^+^, glycine, and the sulfur of a backbone cysteine residue. The phosphorylated pyrimidine moiety is biosynthesized from aminoimidazole ribonucleotide (AIR) via the action of THIC and TH1. THIC contains an iron–sulfur cluster and uses *S*-adenosyl methionine (SAM) in a radical mechanism for catalytic activity. There is evidence for interaction with thioredoxin. To generate the active cofactor, TPP, TMP is first dephosphorylated by a phosphatase (it is not known if this takes place in the plastid or cytosol), and then subsequently pyrophosphorylated by thiamin pyrophosphokinase (TPK). TPP can also be successively dephosphorylated to thiamin by a phosphatase. Expression of the *THIC* gene is regulated through a TPP riboswitch present in its 3′-UTR (depicted as a red line). Abiotic stresses as well as the circadian clock regulate *THIC* and *THI1* gene expression (depicted as green lines). Specific transporter sites across the plastid and mitochondrial membranes are depicted as open barrels.

With a particular focus on genetic engineering, it is clearly imperative to know how thiamin is biosynthesized. Significant progress has been made in this area in plants over the last few years (for comprehensive reviews, see Goyer, 2010; Rapala-Kozik, 2011). Thiamin is formed by the condensation of two separately biosynthesized moieties, hydroxyethylthiazole phosphate (HET-P) and hydroxymethylpyrimidine pyrophosphate (HMP-PP) to form TMP (**Figure 1**). The HET-P biosynthesis pathway in plants is assumed to be similar to that of yeast, in which HET-P synthase (THI4p) catalyzes the formation of the thiazole moiety from NAD^+^, glycine, and a sulfur from a backbone cysteine in THI4p itself (Chatterjee et al., 2011). Homologs of *THI4*, called *THI1* in plants, have been characterized at the genetic level in maize (Belanger et al., 1995), *Arabidopsis* (Machado et al., 1996), and rice (Wang et al., 2006). The pyrimidine moiety is biosynthesized *via* an identical pathway to bacteria in which phosphomethylpyrimidine synthase (THIC) (Raschke et al., 2007) converts aminoimidazole ribonucleotide (AIR) to hydroxymethylpyrimidine phosphate (HMP-P). The latter is then phosphorylated to HMP-PP by a bifunctional protein characterized in maize as THI3 and in *Arabidopsis* as TH1 (Ajjawi et al., 2007a; Rapala-Kozik et al., 2007). The same protein catalyzes the condensation step between HET-P and HMP-PP to produce TMP (Ajjawi et al., 2007a; Rapala-Kozik et al., 2007). All of these steps have been shown to occur in the chloroplast (Chabregas et al., 2001; Ajjawi et al., 2007a; Raschke et al., 2007). As TMP is not directly phosphorylated to TPP, it is assumed to be subsequently dephosphorylated to thiamin by broad specificity phosphatases (**Figure 1**). Indeed, a broad substrate acid phosphatase has been isolated from maize and biochemically characterized showing it can dephosphorylate TMP (Rapala-Kozik et al., 2009). However, the same phosphatase showed relatively higher specificity toward TPP (Rapala-Kozik et al., 2009). Thus, it has been suggested that TPP can also be successively dephosphorylated to thiamin. On the other hand, thiamin can be pyrophosphorylated to make the active cofactor TPP by thiamin pyrophosphokinase (TPK) (Mitsuda et al., 1979; Molin and Fites, 1980; Ajjawi et al., 2007b; Rapala-Kozik et al., 2009; **Figure 1**). It has been suggested that the phosphatase and kinase activities are regulated such that TPP is only provided when the cell needs it (Rapala-Kozik et al., 2009). To date, the subcellular localization of the phosphatase(s) that acts on TMP (or TPP) is unknown. However, the exclusive cytosolic localization of the *Arabidopsis* TPK suggests that the pyrophosphorylation of thiamin into TPP takes place in the cytosol (Ajjawi et al., 2007b).

In prokaryotes, algae, plants, and certain fungi, *de novo* biosynthesis of thiamin is regulated *via* RNA sequences called riboswitches (Winkler et al., 2002; Sudarsan et al., 2003). This mode of regulation is mediated through the direct binding of the product TPP to the pre-mRNA of particular thiamin biosynthetic genes without the need for intermediary proteins. The binding causes a change in RNA secondary structure that interferes with gene expression. A TPP riboswitch sequence has been found in the 3′-UTR of the *THIC* gene of species across the plant kingdom (Sudarsan et al., 2003; Bocobza et al., 2007; Wachter et al., 2007) and in the 3′-UTR of the *THI1* gene in ancient plant *taxa* (Bocobza et al., 2007). In prokaryotes, the change in mRNA secondary structure, induced by metabolite binding, alters transcription termination or translation, whereas in eukaryotes the pre-mRNA is affected by splicing (Breaker, 2011). In contrast, no TPP riboswitch has been found to date in yeast where thiamin biosynthesis is instead regulated through the coordinate action of transcriptional regulators (for review, see Nosaka, 2006; Kowalska and Kozik, 2008).

In this report, we use the current knowledge on thiamin biosynthesis and its regulation in plants to hypothesize on approaches and pitfalls for efficient biofortification of thiamin in crop plants. The hypotheses presented are corroborated by simple feeding studies in *Arabidopsis*, which in turn reveal certain bottlenecks that should be taken into account for biofortification studies.

## IS BYPASSING RIBOSWITCH CONTROL A FAVORABLE WAY TO INCREASE VITAMIN B_1_ CONTENT?

As mentioned above, plants have a TPP riboswitch located in the 3′-UTR of *THIC* that acts as a negative regulator of the thiamin biosynthesis pathway (Bocobza et al., 2007; Raschke et al., 2007; Wachter et al., 2007). The mechanism behind this riboswitch has been studied in *Arabidopsis*. Specifically, supplementation experiments with TPP (1 μM) or thiamin (100 μM) indicates that under such conditions the pre-mRNA of *THIC* undergoes a conformational change that exposes a splice site in the 3′-UTR of this gene (Bocobza et al., 2007; Wachter et al., 2007). As a consequence, there is splicing of intron 2 within the 3′-UTR of *THIC* eliminating the consensus polyadenylation signal and resulting in a transcript that is unstable. On the other hand, in the absence of supplementation there is strong accumulation of an intron retained variant (as the splice site is not exposed). The latter transcript has a single consensus polyadenylation signal that is more stable (Bocobza et al., 2007; Wachter et al., 2007). It is therefore assumed that when TPP reaches high enough levels, regulation of expression of the *THIC* gene occurs through this alternative splicing mechanism, i.e., high TPP levels lead to an unstable mRNA whereas the gene is stably expressed when TPP levels are low. However, the actual levels of TPP required in the cell to induce this splicing event remain to be determined. *In vitro* studies indicate a tight binding constant for TPP to the *Arabidopsis* *THIC* riboswitch (500 nM; Bocobza et al., 2007). As an equilibrium between the different pools of *THIC* transcript would be expected and as TPP levels can reach high micromolar quantities at the cellular level (Ajjawi et al., 2007b; Bocobza et al., 2013), we hypothesize that the TPP binding constant for the riboswitch is likely to be much larger *in vivo*.

As THIC is thought to be one of the key enzymes of the biosynthesis pathway, modulating this regulation would seem to be a tangible approach to increase thiamin content in plants. Indeed, very recently, Bocobza et al. (2013) have studied the impact of the *THIC* promoter as well as its riboswitch on vitamin B_1_ levels in *Arabidopsis*. Interestingly, they could show that *THIC* is regulated in a circadian fashion through its promoter region and that there is an oscillation of the B_1_ vitamer, TMP. Specifically, the *THIC* transcript is highest at the end of the light period and is lowest at the end of the dark period. In addition, the authors introduced a construct harboring the *THIC* promoter, gene and a mutated version of the riboswitch (A515G in the 3′-UTR) that can no longer bind TPP tightly (Bocobza et al., 2007) into a *THIC* knockdown mutant line (Kong et al., 2008). The expectation was that intron 2 of the 3′-UTR would be retained leading to a more stable *THIC* transcript and thereby enhancement of the vitamin B_1_ content. The approach led to an up to threefold accumulation of TMP in leaves. On the other hand, the levels of the cofactor form, TPP, did not show a considerable change and surprisingly, the thiamin vitamer could not be detected. In seeds, an approximate 20% increase in thiamin content was observed in the same lines but no increase in TMP or TPP was noted (Bocobza et al., 2013). However, these transgenic lines exhibited severe chlorosis, growth retardation, and delayed flowering, especially under short day conditions (10 h of light). In another approach, the authors constitutively overexpressed *THIC* in wild-type plants under the control of the *UBIQUITIN1* promoter and terminator. While *THIC* was indeed overexpressed (up to 25-fold) in some of the transgenic lines and a moderate increase in the levels of TMP and TPP was observed (up to 1.5-fold) in leaf material, the corresponding lines were chlorotic (Bocobza et al., 2013). Based on a further comprehensive series of metabolomic and physiological experiments, the authors concluded that the alteration of riboswitch function in this way, while leading to a moderate increase in TPP levels, leads to enhanced carbohydrate oxidation as a result of higher activity of the associated TPP requiring enzymes. Clearly, this has an undesirable impact on the plant manifested as augmented flux through central metabolism and as a consequence negatively perturbing metabolic homeostasis. Therefore, this strategy appears to be unsuitable for vitamin B_1_ biofortification of plants.

## ARE BOTH BRANCHES OF THE THIAMIN BIOSYNTHESIS PATHWAY REQUIRED FOR VITAMIN B_1_ BIOFORTIFICATION?

**FIGURE 2:**
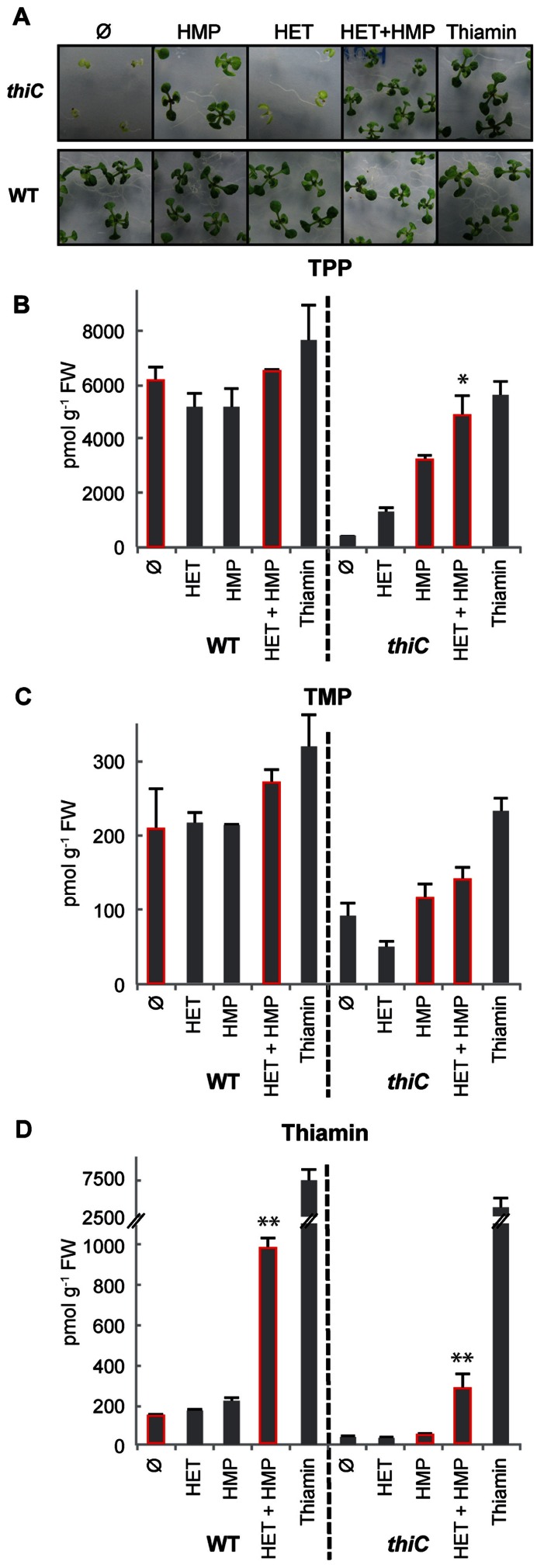
**Effect of feeding vitamin B_1_ precursors on *Arabidopsis***. **(A)** Eleven-day-old wild-type (WT) and *thiC* seedlings (SAIL_793_H10) were grown on half-strength basal salt medium (Murashige and Skoog, 1962) containing 0.5% agar, with or without HMP, HET, HMP and HET, or thiamin (1 μM each, where present). The seeds were stratified at 4°C for 3 days before transfer to a growth chamber maintained under long-day conditions (16 h of light at 150 μmol photons m^-^^2^ s^-^^1^, 22°C; 8 h dark at 18°C). **(B–D)** HPLC analysis of the B_1_ vitamer content (TPP, TMP, thiamin, respectively) in wild-type (WT) or *thiC* after addition of HMP, HET, HMP and HET, or thiamin (1 μM each, where present). In each case, 30 mg of fresh seedling tissue was used. The results shown are the average of three biological replicates. The asterisks denote significant differences between the values of the bars highlighted in red as determined from a two-sided *t*-test, **p < 0.05 and *p < 0.1.

Several studies have demonstrated that thiamin is accumulated in plants during responses to abiotic and biotic stress conditions as well as oxidative stress (Ahn et al., 2005; Ribeiro et al., 2005; Rapala-Kozik et al., 2008; Tunc-Ozdemir et al., 2009). However, few studies have looked at the response of the entire core thiamin biosynthetic genes until recently. Rapala-Kozik et al. (2012) examined the response of *THIC*, *THI1*, *TH1*, and *TPK* (**Figure 1**) under several types of abiotic stress in *Arabidopsis*. We found it interesting that under conditions where upregulation of the expression of the genes was observed, e.g., salt stress, both *THIC* and *THI1* were upregulated to similar relative levels, at least during the initial phases of the response. TPK was also upregulated under the same conditions but to a lesser extent (Rapala-Kozik et al., 2012). Notably in this study, while TPP levels did not show considerable alterations, there were significant changes in the level of the thiamin vitamer, in particular. As the key genes of both the pyrimidine and thiazole branches (i.e., *THIC* and *THI1*) were upregulated concomitant with an increase in thiamin in this study, this prompted us to raise the question whether both branches of the thiamin biosynthesis pathway need to be manipulated in order to increase vitamin B_1_ levels for biofortification purposes in plants. To test this hypothesis, we grew *Arabidopsis* seedlings in the presence of the thiamin precursors from the pyrimidine branch (i.e., HMP) and/or the thiazole branch (i.e., HET). It has previously been demonstrated that both of these compounds are taken up and can restore growth in the corresponding *Arabidopsis* thiamin requiring mutants (Feenstra, 1964). However, as we had the *Arabidopsis* *thiC* knock-out mutant (affected in the pyrimidine branch) in hand (Raschke et al., 2007), we re-confirmed this for HMP. The *thiC* mutant is severely chlorotic and developmentally impaired when grown on basal salt medium (**Figure 2A**, top panels). Supplementation with HMP, HMP + HET, or thiamin in the medium fully rescues the chlorotic and developmental phenotype (**Figure 2A**, top panels). Notably, HMP supplementation alone rescues the phenotype, thus reaffirming its uptake, while HET does not, as would be expected. Neither HMP nor HET supplementation affected wild-type seedling development (**Figure 2A**, bottom panels).

We next quantified the individual B_1_ vitamer contents (i.e., thiamin, TMP, TPP) by high-performance liquid chromatography (HPLC). Similar to other studies on *Arabidopsis* plant seedlings (Rapala-Kozik et al., 2012; Bocobza et al., 2013), TPP was found to be the most abundant vitamer in wild-type seedlings (~6151 pmol g^-^^1^ FW) compared to TMP and thiamin (~207 and 127 pmol g^-^^1^ FW, respectively; **Figures 2B–D**). As expected, we observed that the TPP content increased in the *thiC* mutant when HMP (1 μM) was included in the medium, although the level was not completely restored to that of the wild-type (~3220 pmol g^-^^1^ FW) under these conditions (**Figure 2B**). Interestingly, we observed an additive effect upon supplementation of both HMP and HET to the *thiC* mutant with the TPP levels approaching those of wild-type seedlings under the same conditions (**Figure 2B**). This suggests that HET is indeed taken up, metabolized and converted into TPP by the seedlings. Supplementation with thiamin (1 μM) restores the TPP level in *thiC* to wild-type levels (**Figure 2B**). In the wild-type seedlings on the other hand, supplementation with either HMP or HET alone did not significantly change the B_1_ vitamer content (**Figures 2B–D**). However, when both HMP and HET were added together to the medium, the level of thiamin, in particular, was found to be substantially higher (eightfold increase) from 127 to 973 pmol g^-^^1^ FW (**Figure 2D**). On the one hand, these results suggest that if one precursor is available in excess the other becomes limiting and further implies that bypassing the *THIC* riboswitch alone will not induce B_1_ vitamer accumulation sufficiently. A similar increase in the thiamin vitamer content (sevenfold increase) was observed when both HMP and HET were added to the *thiC* mutant, i.e., from 39 to 272 pmol g^-^^1^ FW compared to HMP alone (**Figure 2D**). These observations provide strong evidence that the first two steps of the thiamin biosynthesis pathway (i.e., those catalyzed by THIC and THI1) are critical in order to increase the thiamin content of plants.

## EXAMINING THE BALANCE BETWEEN THE PHOSPHATASE AND KINASE ENZYMES IN RELATION TO ENHANCING THIAMIN CONTENT

It is interesting that while the thiamin vitamer content was considerably increased by the dual supplementation of HET and HMP, TPP and TMP vitamer contents were not considerably perturbed in wild-type *Arabidopsis* (**Figures 2B–D**). On the one hand, this could suggest that the protein machinery that converts the products of THIC and THI1 into thiamin (i.e., TH1 and a phosphatase) are not limiting for its enhanced production, whereas, the steps necessary for the conversion of thiamin into TPP are. However, as mentioned above, it has been suggested from a study in maize that there is a dynamic equilibrium between phosphatase and kinase actions that interconvert thiamin and its esters depending on the cellular requirement for TPP-dependent enzymes (Rapala-Kozik et al., 2009). To date, it is not known whether the product of the TH1 condensation reaction, TMP, is dephosphorylated into thiamin in the chloroplast, or in the cytosol (**Figure 1**). However, it is known that thiamin is pyrophosphorylated to TPP in the cytosol by TPK of *Arabidopsis* (Ajjawi et al., 2007b). Therefore, we hypothesize that steps restricting the production of the TPP vitamer in particular, could be either the export of thiamin (or TMP) from the chloroplast to the cytosol, the availability of the cytosolic kinase TPK, or dephosphorylation of TPP by a phosphatase. As the transporter(s) for thiamin or its esters out of the chloroplast have not yet been identified, we fed wild-type *Arabidopsis* seedlings with thiamin and followed the production of TPP to perform preliminary tests on the former hypothesis. Significantly, we assume that exogenous thiamin accumulates in the cytosol. Firstly, we could confirm that exogenous thiamin was indeed taken up by wild-type seedlings as shown in **Figure 2D**. Even when the thiamin content was increased 50-fold in wild-type by supplementation (**Figure 2D**), the TPP content only increased by 10% compared to either no supplementation or both HMP and HET supplementation (**Figure 2B**). In a parallel fashion, thiamin addition to *thiC* led to a 92-fold increase of the vitamer (**Figure 2D**), while the level of TPP was similar to that observed under both HMP + HET supplementation (**Figure 2B**). This strongly suggests that a bottleneck for accumulation of the TPP vitamer is either a limitation of the cytosolic TPK enzyme itself or the enhanced action of a TPP phosphatase. It remains to be determined in future studies if TPK activity is a function of its dynamic equilibrium with the associated phosphatase and the requirement of the cell for TPP. Notwithstanding, our feeding studies reveal a fortuitous outcome in the context of biofortification. Dietary vitamin B_1_ is most available to animals in its non-phosphorylated form (Komai et al., 1974; Said et al., 1999). Therefore, specifically increasing levels of TPP in the context of biofortification does not appear to be necessary. Furthermore, in contrast to the findings of Bocobza et al. (2013), where an accumulation of TMP (threefold) and to a lesser extent TPP (1.5-fold) was observed with the strategy undertaken, we do not see a significant increase in either of these vitamers and there is no observable negative effect on the plants under any of the supplementation conditions employed (**Figure 2A**). However, although Bocobza et al. (2013) did not measure thiamin, we do not know if this vitamer was also increased in their study but it is noteworthy that TPK expression was significantly increased with the strategy that was employed. Thus, whether the accumulation of thiamin is due to a limitation in TPK activity or enhancement of a TPP phosphatase, it is a fortuitous act of nature in the context of biofortification. However, any inadvertent increase in the levels of the phosphorylated vitamers through genetic engineering strategies will need to be carefully monitored.

## EXAMINING HOW STRAIGHTFORWARD IT WILL BE TO INDUCE THE PYRIMIDINE AND THIAZOLE BRANCHES

To date, increasing vitamin B_1_ in the context of biofortification has never been overcome in the model plant *Arabidopsis* or in any crop plant. Based on several recent studies, some of which have been discussed above, it is now becoming clear that several regulatory steps will need to be taken into account in order to enhance the vitamin B_1_ content. At its most simplistic level and as shown in this report through feeding studies, genetically engineering the overproduction of the HMP and HET precursors *in planta* should led to an overaccumulation of thiamin. Logically, this could be achieved by overexpressing the THIC and THI1 proteins, respectively. The riboswitch regulation of the expression of *THIC* can be bypassed by employing a transgene construct (i.e., promoter, gene, terminator) harboring a non-functional version of the riboswitch in the 3′-UTR (e.g., A515G) or indeed by employing an alternative terminator as has recently been shown (Bocobza et al., 2013). To counteract differential expression levels, both *THIC* and *THI1* transgenes could be inserted in tandem through a binary vector, under the control of a strong promoter, as has been shown to be successful in the β-carotene (vitamin A) biofortification of rice endosperm (Ye et al., 2000). Moreover, options to use tissue-specific promoters, thus minimizing the energy drain on the whole plant, could be exploited to enhance the process. Clearly, the chosen organ/tissue would need to express the other downstream enzymes of the biosynthetic pathway (i.e., TH1, phosphatases) and the precursors for HMP and HET biosynthesis would need to be available in sufficient amounts in the same tissue. In the context of vitamin B_1_ biofortification in a crop such as rice, the endosperm would be favored but whether the aforementioned requirements are fulfilled in order to engineer such a “minipathway” therein is not yet known.

However, while we surmise that increasing the expression of *THIC* and *THI1* in plants can be achieved, it is important to remember that these are complex enzymes both of which require other key cellular components to fulfill their functions. THIC contains an iron–sulfur cluster that must be coupled with a reductant in order to produce a 5′-deoxyadenosyl radical from *S*-adenosyl methionine that is necessary for its catalytic activity (Raschke et al., 2007; Chatterjee et al., 2008; Martinez-Gomez and Downs, 2008). Moreover, affinity chromatography and proteomic approaches have revealed that THIC could be a target of the thioredoxin/ferredoxin system of oxygenic photosynthesis (Balmer et al., 2003, 2006) and therefore is likely to be also regulated by light (Raschke et al., 2007). On the other hand, while the involvement of THI1 in HET biosynthesis has been deduced from genetic studies (Belanger et al., 1995; Machado et al., 1996), the molecular nature of the substrates required for its biochemical activity in plants remain unclarified. The first study on the substrates of this enzyme claimed that deoxyxylulose 5-phosphate (DXP) is used as one of the precursors for the biosynthesis of HET (Juillard and Douce, 1991), as is the case in microorganisms (Hazra et al., 2009). However, the homolog of THI1 from *Saccharomyces cerevisiae* (called THI4p) has very recently been shown to be a suicidal enzyme (i.e., it can only perform a single turnover upon which it becomes non-catalytic). In this reaction, the thiazole is assembled from sulfur of a backbone cysteine (Cys205) of THI4p itself in addition to glycine and NAD^+^ (Chatterjee et al., 2011). Several key residues including this cysteine are conserved in the plant THI1 proteins. Therefore, a similar mechanism can be anticipated and would represent yet another regulatory checkpoint *via* this enzyme. Furthermore, taken together, the involvement of NADH, the ferredoxin/thioredoxin system and the necessity of active site cysteines additionally make thiamin production highly dependent on the redox state of the cell. Therefore, engineering thiamin production in plants may not be so straightforward with several regulatory checkpoints needing to be surmounted. In this context, any genetic engineering strategy that proposes to enhance production of thiamin will need to be balanced with the physiological impact on the plant. While the branches of the biosynthetic pathway will need to be balanced, the flux through the pathway and its possible draining of other resources necessary for plant health will need to be monitored. These latter statements are corroborated by the undesirable physiology observed in *Arabidopsis* by overexpression of *THIC* alone, as a result of a negative impact on metabolic homeostasis (Bocobza et al., 2013). While the moderate increase in the pool of thiamin esters resulting from the latter strategy may negatively perturb carbohydrate metabolism as suggested by the authors (Bocobza et al., 2013), possible debilitating effects from the overuse of the complex biochemistry associated with THIC also need to be taken into account.

While the thiamin biosynthetic pathway in plants is close to being completely unraveled and could be exploited for biofortification purposes, we must also draw attention to thiamin binding proteins, specific variants of the major seed storage globulins (see Rapala-Kozik, 2011 for review). These proteins have been found and characterized in several types of seeds, e.g., rice, buckwheat, sunflower, and sesame (Golda et al., 2004). They appear to be found in the peripheral cells of the seed, accumulate during seed maturation and are degraded during seed germination (Watanabe et al., 2004). Notably, it is the thiamin vitamer that accumulates during seed maturation and serves as a reserve utilized upon seed germination (Golda et al., 2004). A closer examination of these phenomena could reveal novel strategies for thiamin biofortification of seeds in particular. For example, targeting of a thiamin binding protein to the rice endosperm is a strategy to be explored.

Although it is not the focused topic of this report, exploitation of natural variation is also likely to be a worthwhile approach that should be considered. Recently, Goyer and Sweek (2011) have reported on the identification of wild potato species and primitive cultivars that accumulate high amounts of thiamin compared to a modern potato variety (Russet Burbank). They propose to integrate these materials into breeding programs in order to enhance the nutritional value of potato. A closer investigation of the molecular basis behind the increased thiamin accumulation in these varieties may help genetic engineering strategies in other crop plants.

## CONCLUSION

To date, there is no evidence of toxicity upon supplementation and accumulation of thiamin in plants. This is supported by the feeding studies provided herein. Rather, thiamin has been demonstrated to have beneficial effects enhancing tolerance to both abiotic and biotic stress (Ahn et al., 2005; Ribeiro et al., 2005; Rapala-Kozik et al., 2008; Tunc-Ozdemir et al., 2009). On the other hand, genetic engineering strategies that lead to overaccumulation of thiamin esters must proceed with caution as they may have a negative impact on metabolic homeostasis as indicated by the recent study of Bocobza et al. (2013). A simple engineering of the riboswitch associated with THIC has been discussed at the outset as a likely effective strategy to manipulate thiamin levels in plants, particularly in the context of biofortification. It now appears that the challenge of enhancing thiamin levels in crop plants for biofortification purposes will not be so straightforward. We hypothesize in this study that a balanced provision of the two key precursors (HMP and HET) will lead to the accumulation of the most bioavailable vitamer, thiamin. It will have the added benefit of not being toxic to the plant and could be anticipated to even enhance tolerance to stress. However, genetic engineering of the biosynthesis of the precursor compounds in plants will come with inherent challenges of its own. As discussed above, THI1 and THIC, in particular, are highly complex enzymes and are regulated in a highly complicated fashion, the entire mechanisms of which have not been unraveled yet.

## Conflict of Interest Statement

The authors declare that the research was conducted in the absence of any commercial or financial relationships that could be construed as a potential conflict of interest.
